# Conceptualizing and contextualizing food insecurity among Greenlandic children

**DOI:** 10.3402/ijch.v72i0.19928

**Published:** 2013-05-16

**Authors:** Birgit Niclasen, Michal Molcho, Steven Arnfjord, Christina Schnohr

**Affiliations:** 1 National Institute of Public Health, University of Southern Denmark, Odense, Denmark; 2 Health Promotion Research Centre, NUI Galway, Galway, Ireland; 3 Department of Sociology, University of Greenland, Nuuk, Greenland; 4 Institute of Public Health, University of Copenhagen, Copenhagen, Denmark

**Keywords:** food insecurity, children, Greenland, Arctic, context, concept, HBSC study

## Abstract

**Objective:**

To review the context of food insecurity in Greenlandic children, to review and compare the outcomes related to food insecurity in Greenlandic children, in other Arctic child populations and in other western societies, and to explore the measure used by the Health Behaviour in School-aged Children (HBSC) study.

**Design:**

The study includes literature reviews, focus group interviews with children and analyses of data from the HBSC study. HBSC is an international cross-national school-based survey on child and adolescent health and health behaviour in the age groups 11, 13 and 15 years and performed in more than 40 countries. The item on food insecurity is “Some young people go to school or to bed hungry because there is not enough food in the home. How often does this happen to you?” (with the response options: “Always”, “Often”, “Sometimes”, or “Never”).

**Results:**

The context to food security among Inuit in Arctic regions was found to be very similar and connected to a westernization of the diet and contamination of the traditional diet. The major challenges are contamination, economic access to healthy food and socio-demographic differences in having a healthy diet. The literature on outcomes related to food insecurity in children in Western societies was reviewed and grouped based on 8 domains. Using data from the Greenlandic HBSC data from 2010, the item on food security showed negative associations on central items in all these domains. Focus group interviews with children revealed face and content validity of the HBSC item.

**Conclusion:**

Triangulation of the above-mentioned findings indicates that the HBSC measure of food shortage is a reliable indicator of food insecurity in Greenlandic schoolchildren. However, more research is needed, especially on explanatory and mediating factors.

Food insecurity exists when people do not have adequate physical, social or economic access to food and is the opposite to food security, that is, defined as “a situation that exists when all people, at all times, have physical, social and economic access to sufficient, safe and nutritious food that meets their dietary needs and food preferences for an active and healthy life”.

In 2004, the Greenland Home Rule Government published a national report on food and food policy. The report was meant to provide the basis for formulating a suitable food policy for Greenland. For the first time, it gave epidemiological evidence of food insecurity among Greenlandic children, by revealing that 11% of Greenlandic schoolchildren in grade 6–11 (11–17 years old) reported that they “often” or “always” would go to school or to bed hungry (1). Hunger is considered a more severe type of food insecurity and is defined as the direct sense of the uneasy or painful sensation of not having enough to eat (2). Since then, other investigations have confirmed that a proportion of Greenlandic children face food insecurity (3–6).

Food insecurity is an important child public health problem. In the literature, in Western countries, the evidence also shows that food insecurity in children is associated with a range of adverse effects on health, development and academic performance, and is connected to behavioural and psychosocial problems (7–10). However, food security has not caused much research interest in Greenland, and little has been done to address context, measures, or outcomes of food insecurity in Greenlandic children. The data in the cited report came from a national school survey, the Health Behaviour in School-aged Children (HBSC) study. It is the only tool that has been used repetitively to address food insecurity in Greenlandic children. Addressing the validity of the tool is therefore highly important. This can only be done through comparison of the context, prevalence and associations of food insecurity among Greenlandic children to findings in other Western and other Arctic countries as well as by exploring the instrument itself. To do so, different methods and data sources need to be included and the results triangulated. The rationale behind the triangulation in this study is: If the context of food insecurity in Greenlandic children resembles that in other indigenous Arctic populations; if the measure of food insecurity has content validity; and if the associations of food insecurity in Greenlandic children found with the measure used in the HBSC survey are similar to those found in other indigenous Arctic and Western child populations, then the HBSC item on food insecurity must be considered valid.

With this background, the aims of this study was: (a) to review the context of food security and insecurity in Greenlandic and Inuit or Aboriginal children in other Arctic countries; (b) to give the prevalence of food insecurity in children in Arctic countries with an Inuit population and comparing food insecurity in these countries and Denmark using the HBSC item; (c) to review associations to food insecurity found in indigenous Arctic child populations, in child populations in other Western countries, and to reporting hunger using the HBSC measure; and (d) to explore the validity of the HBSC measure on food security used in Greenland.

## Materials and methods

The research design included literature reviews, focus group interviews and analyses of data from the international and the Greenlandic part of the HBSC study. HBSC is an international cross-national school-based survey on child and adolescent health and health behaviour in the age groups 11, 13, and 15 years. The survey collects comparable data on health and health behaviour in more than 40 countries and regions in Europe, North America and Israel, every 4 years (11). More than 200,000 children took part in the most recent survey in 2010. In the Greenlandic part of the HBSC survey, all schoolchildren aged 11–17 (grade 5–10) were included. Data from the 2010 survey included 2,254 students corresponding to 40% of all Greenlandic schoolchildren in the included grades. The school response rate in the study was 65% (6).

The HBSC item on going to bed hungry, or to school, was added in the international HBSC survey in the 2001/2002 study in acknowledgement of the fact that HBSC lacked a measure of very low socio-economic status. However, by wording, the HBSC item measures food insecurity, and not poverty. The issue is: “Some young people go to school or to bed hungry because there is not enough food in the home. How often does this happen to you?” (With the response options: “Always”, “Often”, “Sometimes”, or “Never”).

*Aim I: To review the context of food security and insecurity in Greenlandic and Inuit or Aboriginal children in other Arctic countries:* A literature search in PubMed on “Greenland” and (“food security” or “food insecurity”) and (child*) was performed. The search had 3 hits, 1 was relevant (12). Another search on “Greenland” and (“diet” or “food security” or “food insecurity”) was performed; it had 248 hits and 4 were relevant (13–16). An Internet search on recent books on Inuit Health, national research reports and official documents from the National Documentation Centre on Children, the National Institute of Public Health, the Government of Greenland and the Health Care System provided 7 reports and books (1, 3–6, 17, 18). In addition, a search in PubMed was made on (“indigenous” or “aboriginal”) and (“Canada” or “United States” or “Russia”) and (“food security” or “food insecurity”) and “child”*, limitations: Humans, English, All Child: 0–18 years. The search returned 130 hits, of which 58 were selected for further reading and 8 were found relevant (19–26). A search on national reports and other papers on the Internet on Inuit yielded 5 publications (27–32).

*Aim II: To give the prevalence of food insecurity in children in Arctic countries with an Inuit population and compare food insecurity in these countries and Denmark using the HBSC item*. The publications found in the above-mentioned searches were reviewed for data on frequencies. Data from the international 2009/2010 HBSC investigation were utilized to give the national prevalence of food insecurity in Greenland, Canada, United States, Russia and Denmark. Denmark was included because Greenland is a former Danish colony and has a social organization resembling the Danish.

*Aim III: To review associations to food insecurity found in Greenlandic and indigenous Arctic child populations, in child populations in other Western countries, and to reporting hunger using the HBSC measure:* A search in PubMed was made on (“food security” or “food insecurity”) and child*. Limitations included: Humans, English, All Child: 0–18 years. This search yielded 391 hits. A total of 49 reviews were selected; after reading the summaries, 5 reviews (7, 9, 26, 27, 33) and 2 other papers (8, 10) were selected. A second literature search was performed in PubMed with the limitations: Humans, English, All Child: 0–18 years with limitations: English on (“hunger” or “hungry” or “food poverty”) and “HBSC”. The PubMed search yielded 4 hits, 2 of which were used (34, 35). On the Internet, 3 additional publications were found (36–38). For associations in Greenlandic children: The search in Aim I was used. During the review on the associations to food insecurity, it was revealed that they could be grouped into only 8 domains, namely: (a) Dietary consumption and nutritional outcome; (b) Physical health and development; (c) Life satisfaction and mental health; (d) Behavioural and psycho-social functioning; (e) Academic performance; (f) Risk factors in the home environment; (g) Health behaviours; and (h) Socio-demographic risk factors.

*Aim IV: To explore the validity of the HBSC measure on food security used in Greenland. The aim was divided into 2 sub-units: Aim IVa) to explore the content validity of the HBSC item, and Aim IVb) to explore outcomes associated to food insecurity in the HBSC study, compared to associations found in other studies. Aim IVa:* To explore the content validity of the HBSC item in Greenlandic schoolchildren, 2 group interviews with children in grade 9 (aged 14–15 years) in a public school in Nuuk were analyzed. The interviews were carried out in Greenlandic and taped. The same interview guide, including questions about understanding of the question, thoughts about the question and its content, was used. The interviews were transcribed and translated into Danish by the interviewers. Analyses were performed following Kvale's work (39). *Aim IVb:* Data from the Greenlandic part of the HBSC study in 2010 were used to analyse if the associations of food insecurity reported in other studies (aim III) were retrievable using the HBSC item. For the 8 domains revealed under Aim III, the following items were analyzed: (a) *Dietary consumption and nutritional outcome:* “Eating breakfast on all schooldays” and “Eating fruit and drinking soft drinks nearly daily (5–6 times a week or more)”; (b) *Physical health and development:* “Physical symptoms (headache, stomach ache or back ache) nearly every week” and “Good or excellent self-rated health”; (c) *Life satisfaction and mental health:* “Psychical symptoms (depressed, irritable or nervous) nearly every week” and “Having the best possible quality of life”; (d) *Behavioural and psycho-social functioning:* “Never been bullied”, “Never bullying others” and “Feel accepted by classmates”; (e) *Academic performance:* “Good or very good academic achievement” and “Low pressure from schoolwork”; (f) *Risk factors in the home environment:* “Difficult to talk to mother or father about something that really bothers” and “Eating dinner with parents every day”; (g) *Health behaviours:* “Tooth brushing twice a day”, “Daily smoking” and “Have been drunk more than once”; and (h) *Socio-demographic risk factors:* “Age” (11–12 years old/13–14 years old/15–17 years old), “Gender” (boy/girl), “Low family affluence” (proxy for high/low economic access) and “Living in a settlement” (proxy for living remote). The exact wording of the item questions in English can be found in the International HBSC Study protocol (http://www.hbsc.org), and in Danish or Greenlandic in the national reports (5, 6). Analyses were made in SPSS 17.0 using Chi-square test.

## Results

### The context of food security and insecurity in Greenlandic and Inuit or Aboriginal children in other Arctic countries

Language and diet are central to the circumpolar Inuit culture (18). In general, the major challenges to food security found were very similar among Inuit across the Arctic region and were associated to a westernization of the diet, contamination of the traditional diet, economic access to healthy food and socio-demographic differences in having a healthy diet (16).

All Arctic countries have made nutritional guidelines for their Northern populations. These guidelines take the potential health impact of contaminants into consideration and most countries have special guidelines on the consumption of traditional foods regarding children. However, concerning imported foods, guidelines in general follow international recommendations (14).

The traditional Inuit diet was mainly based on marine mammals, but also on local birds, fish and land-based animals. The high use of imported food marks the globalization of the diet in Arctic indigenous populations. It has meant a lower consumption of domestic foods and an increase in the consumption of sugar and processed foods, but also of vegetables and fruit. Traditional foods are important sources of vitamins and other nutrients. As opposed to imported food, domestic foods are often self-supplied (15, 16, 18).

The contamination of the traditional diet is highly important to food security. As the top predator in the food chain, Inuit who consume marine mammals are at high risk of bioaccumulation of contaminants. In children, the pollutants have potential negative effects on their neuro-physiological development, on certain hormones and on their immune system (16).

Currently, imported foods provide 75–80% of the energy consumed in adult Greenlanders, while no information exists in relation to energy consumption in children (12). No data on energy distribution were found from Canada, United States, or Russia. Across the Arctic, children and young people are eating less traditional food than the older part of the populations (12, 15, 18, 23). Eating regular meals is not a tradition in the Inuit population (15). It has been discovered that Inuit and other remote Northern populations have a risk of having less healthy dietary choices (15, 23). Greenlandic children in the settlements eat more traditional food, but also have a higher consumption of candy and a lower consumption of fruit, than children in towns (5, 12). Economic access to healthy food and a variation of imported food are important to food security in the Arctic. The variation of imported food available in the North varies among countries and regions, but generally the highest availability and the lowest prices are seen in towns, compared to more remote areas (15, 24, 29, 31). Prices on many basic foods are high, compared to the available economic resources within many Inuit families (13, 24).

The policies to address the issue of economic access and variation differ highly among Arctic states. In Greenland, the provision of basic healthy food is considered to be a governmental responsibility. Healthy foods are subsidized and made available to the population in remote areas through publicly owned stores. It secures basic foods to prices resembling town level, even if the variety of basic foods provided depends on the actual population size. As a consequence of the reported food insecurity in schoolchildren, the government offered the municipalities to share the financial costs for offering a daily meal to all children in public schools. In Canada and the United States, a more complex pattern of subsidized market mechanisms and charity exists (32).

### The prevalence of food insecurity in children in Arctic countries with an Inuit population and comparing food insecurity in Arctic countries and Denmark using the HBSC item

Investigations into the prevalence of culturally related food insecurity regarding traditional foods, or on food insecurity in children measured in the households (child food insecurity), have not been undertaken in Greenland, as opposed to Canada and the United States. In Greenland, the only data sources on the prevalence of food insecurity found were regarding food shortage (hunger) either using self-reported data from schoolchildren or parental reports in 0–14 year-olds (1, 3, 5, 6, 17). Nearly all studies in other Northern populations have used data on parental report either on household food insecurity or on household food insecurity in households with children. Most studies in the United States have used the United States Department of Agriculture (USDA) Measurement of Food Insecurity Scale, while most Canadian studies have used a slightly modified version of the USDA scale made by Health Canada (20, 21, 23, 28, 40). This makes comparisons difficult, especially as none of the found studies have combined issues on parental reports on household or child food insecurity, and self-report in schoolchildren.

Self-reported data from the Greenlandic HBSC study revealed that in 2002, 7% of 11–17-year-old schoolchildren reported that they *always* went to bed or to school hungry, because there was no food in their home, and an additional 4% stated that it happened *often*, while the proportion that *never* went to school or to bed hungry was 69% (1, 17). In both 2006 and 2010, 17% reported experiencing going to bed hungry *always* or *often*, while 59% in 2006 and 68% in 2010 reported it *never* happened to them (5, 6). In 2002 and 2006, Greenland had the lowest proportion of children reporting *never* going to bed or to school hungry among the HBSC countries (6). Also, in Northern Canada, self-reported food insecurity in schoolchildren using the HBSC item is high. In Yukon, the highest proportion of children going to bed or to school hungry *sometimes, often or always* was found in rural boys in grade 6–8 (43%) and the lowest in rural girls in grade 9–10 (23%) (38), thereby reporting figures close to the Greenlandic ones.

In Greenland, data based on parental reports suggest that among 0–14 year-olds, 3% of the children had experienced going to bed hungry, because there was no food in the home, and a total of 5% was reported to have suffered neglect (3). In contrast, many studies from Canada and the United States using the USDA measure or a variation of fit, indicate that Northern and indigenous populations experience food insecurity more often than the general population and that children are at special risk (20, 24, 27, 28, 40). In 2004, Canadian aboriginal households were 2.6 (95% CI 2.1–3.2) more likely to report food insecurity compared to non-aboriginal households (40). In Nunavut, 49% of households reported not having enough to eat *often or sometimes* during the year prior to the study, compared to 7% for overall Canadian households (19). In Inuit households with children, an even higher prevalence of food insecurity has been found. In 2007–2008, 31% of Inuit children in 16 Nunavut communities lived in moderate child food insecure homes, while another 25% lived in severe child food insecure homes (20). In Alaska, 27% of households with children and 19% of all households were food insecure, compared to a mean 19 and 16% in households in the United States, respectively (28). In 2006–2008, 23% of Alaskan Indian/Alaskan native households were food insecure (22). No data were found for Arctic Russia.

Analysing HBSC data (Table I) did confirm that Greenlandic children report food shortage to a higher extent than children in Denmark and in other Arctic countries with an Inuit population. In 2010, Greenland had the highest percentage of children in the Arctic among 11 and 13 year-olds reporting going to bed or to school hungry *always or often*, while in the 15 year-olds, Greenlanders were second only to Danes (3.5% vs. 4.5%). For *always*, the proportion in the 11-year-old Greenlanders was nearly 10 times the average of HBSC countries, while the 13 and 15 year-olds reported about 4 times the HBSC average. In all age groups, Greenlandic children had the lowest proportion reporting *never* going to bed or to school hungry.

**Table I:** Percentage of children age 11, 13 and 15 years in Greenland, Denmark, Canada, the United States and Russia reporting to go to bed or to school hungry, because of lack of food in home (data from the International HBSC study)

%		Always (%)	Often (%)	Sometimes (%)	Never (%)
Greenland (n=1,082)	11 year olds	11.1	8.4	16.5	64.1
	13 year olds	5.8	7.4	18.0	68.8
	15 year olds	3.5	4.9	20.0	71.6
Denmark (n=3,990)	11 year olds	6.0	2.1	13.4	78.4
	13 year olds	4.0	1.6	13.6	86.7
	15 year olds	4.5	2.0	11.9	81.7
Canada (n=15,578)	11 year olds	1.1	3.1	26.1	69.6
	13 year olds	1.2	2.5	21.2	75.1
	15 year olds	1.3	3.3	19.6	75.8
US (n=6,170)	11 year olds	1.8	3.4	24.0	70.2
	13 year olds	1.4	3.6	21.2	73.9
	15 year olds	1.4	3.0	29.2	76.4
Russia (n=4,946)	11 year olds	3.6	4.9	13.1	78.4
	13 year olds	3.6	5.2	13.3	77.8
	15 year olds	2.8	5.4	13.3	78.5
Mean in all HBSC countries (n=208.203)	11 year olds	1.5	2.2	17.1	79.2
	13 year olds	1.2	2.1	15.2	81.5
	15 year olds	1.2	2.4	14.3	82.1

The HBSC samples are national samples. The comparison cannot take the inter-country variation found by other methods into consideration. The data from Yukon (38) indicate that comparison only between Inuit or Arctic populations might present figures much more similar to the Greenlandic ones.

### Associations to food insecurity found in indigenous Arctic and Greenlandic child populations, in child populations in other Western countries, and to reporting hunger using the HBSC measure

In indigenous Arctic populations and other Western child populations, the relationships between food insecurity and children's health, behaviour and development seem to vary according to the child's age, gender and ethnicity (27). As stated above, the literature on associations to food insecurity could be grouped under only 8 domains: (a) Dietary consumption and nutritional outcomes (7, 9, 20, 21, 23, 40); (b) Physical health and development (7–9, 33); (c) Life satisfaction and mental health (7, 9); (d) Behavioural and psycho-social functioning (7, 9); Academic performance (7, 9, 10, 31); (e) Risk factors in the home environment (9, 23, 40); (f) Health behaviour; (g) Socio-demographic risk factors such as poverty or living remotely is highly associated to food insecurity and children are the age group most likely to live in food insecure households (19, 23, 24, 27–31, 41–43). The specific findings are summarized in Table II.

**Table II:** Domains and associations to food insecurity in children in Arctic aboriginal and Western countries reported in the literature

	In Western children	In Inuit and Indigenous children in the Arctic
Dietary consumption and nutritional outcomes	Consume fewer servings of fruits and vegetables (9) Consume fewer milk products Nutrient inadequacy for protein, micro-nutrients (vitamins, magnesium, phosphorous, and zinc) (7, 9, 33) Iron-deficiency anaemia (7) Overweight/obesity (conflicting evidence) (9, 42) Under nutrition or underweight for age and height (7, 9) Stunting (7, 9) Breastfeeding seem to give some protection (7) Report eating crisps, fried potatoes and hamburgers more often (schoolchildren) (35) Less likely to report eating breakfast on schooldays (schoolchildren) (37)	Lower median Healthy Eating Index (21) Consumed a lower mean number of different food items in the past day (21) Lower prevalence of any milk consumption (21) Higher median consumption of high-sugar drinks (21) Higher prevalence of traditional food consumption (preschool children) (21) Went hungry or skipping meals (20) High consumption of high-sugar foods and drinks in the household (23) Low consumption of grains, vegetables, fruits and milk in the household (23)
Physical health and development	Children having their health reported as “fair/poor” by parents (7, 9) Less children report excellent health (schoolchildren) (36, 37) Increased rates of acute and chronic illness (9) Predictor of chronic illness (preschool and schoolchildren) (7) Greater odds of hospitalization since birth (in toddlers) (7, 9) Low physical function (in 3–8 year-olds) (9) Being in developmental risk (infants and toddlers) (7) Parental developmental concern (9) Poor developmental trajectories (from kindergarten through 3rd grade) (9) More frequent stomach ache, head ache and colds (7, 9) More frequent report of physical symptoms (schoolchildren) (37) Lower IQ (partly explained by mothers personality and insensitivity to the children's needs) (8) Have been injured more often (schoolchildren) (37)	Less likely to have their health reported as excellent/very good (preschool children) (20) Less likely to be obese (23) Overweight/obese (29)
Life satisfaction and mental health	Increased risk of emotional problems (including higher levels of anxious and irritable, aggressive and oppositional behaviours) (schoolchildren) (9) Having increased anxiety (7) More likely to be aggressive (7) Having increased internalizing behaviour scores (7) Lower health related quality of life (9) Increased risk of having seen a psychologist (schoolchildren) Increased rate of depressive disorder and suicidal symptoms (in adolescents) (7) More likely to have clinical level of psychosocial dysfunction (6–12 year-olds) (7) Behavioural problems (partly explained by mothers personality and insensitivity to the children's needs) (8) More frequent report emotional symptoms (schoolchildren) (37) Less likely to report being happy with their lives (schoolchildren) (37)	
Behavioural and psycho-social functioning	Lower psychosocial functioning (9) More likely to have seen a psychologist (7) Lower behavioural functioning (9) More difficulty with getting along with other children (teenagers) (7, 9)	Negative impact on psychological development (20)
	Having a decline in social skills (among boys) (7, 9) More often suspended from school (teenagers) (7, 9) More likely to have had dystonia/thoughts about death/attempted suicide (7) Higher level of hyperactivity, absenteeism and tardiness (7) Higher emotional stress (8) More likely to report poor communication with parents (schoolchildren) (34, 37)	
Academic performance	More likely to have repeated a grade (7, 9) Receive lower arithmetic scores (7, 9) Impaired academic performance in reading and mathematics (9) Smaller increase in reading scores (7)	Negative impact academic performance (20)
Risk factors in the home environment	Stressful life events (7–9) Chronically ill parent (9) Recently divorced parent (9) A child with physical disability or injury, learning disability or physical disability (9) Medical care (no usual source of care, postponed care, postponed medications and not receiving the recommended well-child care visit) (with simultaneous housing instability) (9) Smoking in the household (9) Parental depression (especially maternal) (7–9) Parental practices, attachment and proficiency (toddlers) (9) Substance abuse Lower interviewer perceived household sensitivity to children's needs (8)	Substance abuse (19)
Health behaviour	More likely to report high level of television viewing (schoolchildren) (34, 37) More likely to have a history of drunkenness (schoolchildren) (34, 37) More likely to smoke (schoolchildren) (34, 37) More likely to have infrequent tooth-brushing (schoolchildren) (34, 37) Less likely to report to use seatbelt (schoolchildren) (34, 37)	
Socio-demographic risk factors in the household	Poverty/low income in household (7–9, 27) Minority/immigrant status/indigenous (7, 9) Low level of educational achievement in parents Unemployment or low level of labour force participation Getting social assistance/income support (27) Lone parent family (7, 27) Household headed by a woman (7, 27) Public or rented housing (7) Multiple child household or crowding Households with young children (7) Living remote (29)	Aboriginals including Inuit (20, 27, 40) Poverty or be in the lowest income adequacy category (27, 40) Multichild households (3 or more) (40) Large families (19) Household crowding (21, 23) Having a home in need of major repairs (21, 23) Public housing (21, 23) Low levels of education achievement in parents (40) Reliance on social assistance and welfare (21, 40) Low level of labour force participation or income from sources other than wages or salaries (40) Households headed by lone parents (19) Families who have no active hunter (19, 23) Low cash-flow (19)
		Number of adults in the home showed tendencies for a protective effect (21) Lack of economic access (20)
Socio-demographic risk factors in the child	Age (schoolchildren) (36, 38) Gender (schoolchildren) (36, 38) Living remote (38)	Age (schoolchildren) (16) Gender (schoolchildren) (16) Living remote (schoolchildren) (16)

In Greenland, only one other data-source than the HBSC study had analyzed associations to food insecurity in children. It is a study on the effect of poverty that interviewed parents and children in families receiving social benefits (4). Parents in these families expressed often to be *short on money before payday and that their children were aware of the situation, especially if short on food*. The children expressed that they sometimes *did not get enough to eat at home or did not bring enough food for lunch in school*. They expressed *complex strategies from giving up, lower their expectations, eat at family or friends or get a job*. The motivation to get a job was connected to wanting to help the family economy and for them to be able to cover their own needs (4).

In the reports from the Greenlandic HBSC study, *risk factors in the home environment* found included: *mother tongue* as 65% of Greenlandic and 83% of Danish speaking children *never* had experienced going to bed or school hungry because of lack of food at home (17); *socio-demographic risk factors* found were *age* as less of the 11–12 year-olds *never* experienced hunger (5, 6); *gender*, as more boys than girls were affected (5, 6, 17); *wealth*, as 38% of children who considered their family wealthy never went to bed hungry compared to 70% in wealthy children (17); and *living in a remote area* as the proportion going to bed or to school hungry was higher in settlements than in the capital (5, 6, 17).

Associations to reporting hunger using the HBSC measure in other countries have revealed that children reporting going to bed hungry are also *less likely to eat fruits, vegetables and brown bread*, are *more likely to eat crisps, fried potatoes and hamburgers*, are *less likely to eat breakfast on schooldays, and eat dinner on weekdays* (35), are *less likely to report excellent* health and *feeling happy about their lives*, and are *more likely to report frequent emotional and physical symptoms* (37). Additionally, they are found more likely to report poor *communication with parents, and high levels of television viewing*, have a *history of drunkenness*, to *smoke*, and have *infrequent tooth-brushing* and *non-use of seatbelts* (34, 37). Also *socio-demographic risk factors* such as *age, gender and low family wealth* have been found associated to reporting hunger (35, 36, 38) (Table II).

### Explore the validity of the HBSC measure on food security used in Greenland and compare outcomes on central items associated to food insecurity between Greenlandic children reporting having and never having experienced hunger

It was found that the children interviewed understood the question as it was intended. They stressed socio-demographic factors as important to not having enough to eat, but also factors within the family such as, neglect and parental alcohol abuse. They understood the question as “hungry in the daily life” and “wanting to eat”, but not as starving, and described the symptoms of hunger and connected hunger to a wish to have something to eat. The respondents named children who were poor, who have experienced neglect, who live in settlements or are homeless, or whose parents had used the money for other things, for example, on alcohol. The mentioned reasons for going to bed hungry included not having had time to eat, experiencing sadness and therefore not wanting to eat, and to being ill because of not having had anything to eat. They expressed that some children might come to school hungry if they did not have time to eat, but also “that the parents do not tell that there is food, because they have used the money for alcohol”. The interviewed did not connect hunger to wanting to lose weight. It is concluded that the focus group interviews demonstrate content validity of the HBSC item although some children seem not to take notice of the last part of the item question: “because there is not enough food in the home”.

Comparing outcomes on central items associated to food insecurity between Greenlandic children reporting having and never having experienced going to bed or to school hungry, because there was no food at home, was performed on the above-mentioned items and shown in Table III. Food insecurity was found highly significantly associated to poorer outcomes on one or more of the analyzed items on all 8 dimensions. The largest differences between children having or never having experienced hunger were found for the socio-demographic risk factors analyzed: *Age, gender, low family affluence* and *living in a settlement* (p<0.001 for all). However, for Dietary consumption and nutritional outcomes (*Eating breakfast on schooldays*); Physical health (*Good or excellent self-rated health*); Behavioural and psychosocial functioning (*Never been bullied* and *Never bullied others*); Academic performance (*Good or very good academic achievement* and *Not pressured by school work*); Risk factors in the home environment (*Eating dinner with parents*); and Health behaviours (*Tooth brushing twice a day*), highly significant differences were found. Also, on *Eating fruit 5–6 times a week or more*, *Physical symptoms* (*headache, stomach ache or back ache*) *nearly every week* and *Having the best possible quality of life* significant differences were found, while with regard to *Drinking soft drinks*, *Psychical symptoms*, *Feel accepted by classmates*, *Talking to their father*, *Daily smoking* and *Being drunk more than once*, no significant associations were found (Table III).

**Table III:** Comparison between schoolchildren age 11–17 who “never” and “sometimes, often and always” experience to go to bed or to school hungry at central HBSC items on the 8 domains

Area	Item	Never	Sometimes, often or always	Chi^2^
Dietary consumption and nutritional outcomes	Eating breakfast on all schooldays	64.7	52.9	p<0.001
	Eating fruit 5–6 times a week or more	29.4	23.9	p=0.02
	Drinking soft drinks 5–6 times a week or more	46.9	46.3	p=0.82
Physical health and development	Physical symptoms (headache, stomach ache or back ache) nearly every week	66.5	61.3	p=0.04
	Self-rated health “good” or “excellent”	83.7	77.0	p<0.001
Life satisfaction and mental health	Psychical symptoms (depressed, irritable or nervous) nearly every week	59.5	56.2	p=0.21
	Very best quality of life	33.6	28.0	p=0.02
Behavioural and psycho-social functioning	Never bullied	64.2	56.7	p=0.002
	Never bullied others	59.5	50.7	p<0.001
	Feel accepted by classmates	71.1	74.2	p=0.19
Academic performance	Good or very good academic achievement	71.5	62.9	p<0.001
	Not pressured by schoolwork	40.7	31.9	p<0.001
Risk factors in the home environment	Difficult to talk to mother about something that really bother	60.2	52.6	p=0.003
	Difficult to talk to father about something that really bother	43.0	42.5	p=0.83
	Eating dinner with parents every day	75.5	67.9	p=0.001
Health behaviours	Tooth brushing twice a day	65.7	50.6	p<0.001
	Daily smoking	24.8	28.1	p=0.13
	Been drunk more than once	22.2	20.9	p=0.52
Socio-demographic risk factors	Age (see Table I)			
	Gender, boys/girls experienced hunger	62.3/73.5	37.7/26.5	p<0.001
	Low family affluence	34.0	47.3	p<0.001
	Living in a settlement	18.4	28.4	p<0.001

## Discussion

The background on which food security in Arctic populations must be evaluated is the rapid dietary transition (16). The shift to a more modernized economy has introduced competition between traditional food consumption and a more westernized diet (29). The same development is found in other populations that have been through rapid social transition. As an example, urbanization is accompanied by the same social and economic trends, which encourages a food supply higher in fat content, sugar and salt, lower prices of processed foods relative to stable foods, and exposure to corporate food distributors (44).

Food and eating have important social, cultural and symbolic functions. What we eat is “a sign of membership”, social status and spiritual worth (26). Therefore, the westernization of the Inuit diet is also connected to changes in cultural identity. As Inuit youth and children generally eat less traditional food than the older generations, time might accelerate this development (12, 15). Another important accelerator of lesser consumption is the contamination of a major resource in the traditional diet: the marine mammals. This contamination has made it necessary to provide special guidelines on the consumption of traditional foods, with regards to children, and has raised concern and insecurity in the general population regarding safe consumption of traditional foods.

Food insecurity was found to be associated with economic access to healthy foods and to socio-economic factors. However, the relation between traditional food consumption, food insecurity, poverty and socio-demographic factors is complex. The reviewed studies indicate that food insecurity in children in both Greenland and Canada is connected to a higher consumption of traditional foods, but also to a higher consumption of soda and junk food, as well as having less variation in the diet (12, 21). This somewhat contradictory finding might be explained by a shared determinant: the socio-demographic conditions. Both a high consumption of traditional foods and having a less than healthy diet are associated to living remotely and being less wealthy, and in adults also to lower education and lower socio-economic position, which again is associated to live in a remote setting (12, 18, 21, 23).

At the population level, socio-economic differences in having a healthy diet are found (16), and low income is undeniably the single greatest risk factor for household food insecurity in the Arctic as well as worldwide (31, 45, 46). The coexistence of household food insecurity with poverty, and the well-documented association between poverty and ill health, makes it difficult to identify health consequences other than dietary inadequacies that are specific to food insecurity (30). At the individual level, poverty is neither a specific nor a sensitive indicator of food insecurity (31, 45). Poverty or limited household resources only provide a crude indication of the available resources for food within a household at any given point (45). This is also true in Greenland, as the interviewed children pointed out: Available resources might not be used for food. Both the analysis and other data have confirmed that food poverty is not restricted to children from lower social class families (17, 27, 35).

Without doubt, food insecurity in children exists in Greenland too. However, it has been sparsely investigated, and only data on more severe food insecurity measured as hunger were found (3–6, 17). The proportion of food insecure children found varied widely, from 3% in 0–14 year-olds reported by the parents (3), to 11–17% reported by 11–17 year-olds themselves (5, 6, 17). There are many reasons for this variation. The first is that different survey instruments were used. The second is the difference in the age of the children investigated. Younger children are generally protected from disrupted eating patterns and reduced food consumption at much greater levels of adult food insecurity than older teenaged children (41). The third is the difference in the informer. Research on food insecurity in children often relies on parental, particularly maternal, reports on children's experiences. According to mothers’ narratives, when food becomes scarce, the mother employs a sequence of strategies to manage increasingly severe situations with an overall aim of protecting children from hunger (47). Still, food insecurity in children is a sensitive issue associated with social stigma, and therefore might be underreported by parents not least in an affluent society. As it has been expressed, there is a disjuncture between the perspective that most U.S. children are protected from food insecurity and substantial research showing that children experience negative developmental outcomes when they live in food-insecure households (48). Christensen et al. (3) themselves questioned the low prevalence of food insecurity found in their study as other reports on child health as well as police files have revealed higher proportions of different aspects of child neglect.

In fact, children are often better reporters than their parents of their own experiences of internal state. Greenlandic children in poor homes expressed many of the same coping strategies on food insecurity as expressed by, for example, U.S. children (4, 48). Their conceptualization involved components of awareness and responsibility grounded in their experiences, such as worries about parental stress and hardships, feelings of anger and helplessness, and cognitive vigilance to monitor the household food situation when parents are trying to hide what is happening (48). In accordance with this, validation work by the HBSC study has shown that children reports are generally reliable (49). Basically, the children's own report of their perception of going to bed or to school hungry must be believed. Although, the interviews somewhat indicated that not all children take notice of or understand the last part of the HBSC item (“because there is not enough food in home”). In some children, their hunger may not be due to lack of food in the home.

The HBSC study in Ireland found the relative odds of hunger somewhat connected to family management. Similarly, in Greenland an association between food insecurity and the home environment has been speculated (5). These indications that food security is related not only to family wealth but also to family function are important to consider. Characteristics of parents and households that affect children's development might also contribute to determining whether children's households become food insecure. Neglectful and chaotic households are associated with parental factors linked to food insecurity, including poor parental mental health, substance abuse, cognitive impairment and limited social support (8). If cognitive, behavioural and emotional problems among food-insecure children share a common cause with food insecurity, interventions addressing children's food situations will fail to fully ameliorate poor developmental outcomes (8). Thus, a variety of social, economic, political and environmental factors must be considered when addressing food insecurity through policy instruments.

The prevalence of food insecurity seems to vary widely around the Arctic Circle. The different measures on food security used in Greenland, Canada and the United States indeed make direct comparisons difficult. Only one study with Greenlanders has used the modified USDA scale and thereby can be compared to Canadian data (13). It found that less than 10% of adult Greenlanders in a small town on the West Coast suffered food insecurity. This is much lower than figures found among Inuit in Northern Canada. A considerable intra-country variation and a North–South gradient seem to exist in Canada and the United States. A consistent finding within Canada and the United States is that aboriginal populations especially in the North are at much higher risk (19, 20, 22, 23, 28). In Greenland, Greenlandic-speaking children compared to Danish-speaking children are at somewhat higher risk of food insecurity (17). It is not possible to take the intra-country variation between Arctic HBSC countries into consideration in our analyses, which probably is one of the reasons why Greenland had the highest proportion of children never going to bed or to school hungry (Table I). However, when comparing with Yukon, figures in schoolchildren were comparable to findings in Greenland (38).

Because of the complex, multidimensional nature of food insecurity and the strong subjective element of the construct, it is difficult to identify a simple “gold standard” against which food insecurity scales can be validated (30). Using only one item to provide an indication of food insecurity, the HBSC measure represents a “red flag” approach to measuring food security in schoolchildren. A “Red flag” approach can provide important information about food insecurity (31), but does not yield meaningful data on the full range of elements in the construct or the severity of the problem; nor does it facilitate comparisons of results across surveys. As stated previously, a key element in the validation of the HBSC item is to analyze if the associations with food insecurity in Greenlandic children found with the HBSC item are similar to those found in the literature. As summarized in, many associations between food insecurity and negative outcomes of children's health, development and well-being were reported. To explore if the HBSC measure was associated with negative outcomes on the same domains, key HBSC items on each of the domains were compared between children who never had and children who had experienced food insecurity sometimes, often or always. It showed that food insecurity measured with the HBSC item was associated with highly significant negative outcomes on one or more key items on all domains (Table III). The finding is consistent with analyses on Irish HBSC data, where children reporting having experienced hunger had higher risk of somatic and mental symptoms, negative health perceptions and lower life satisfaction (35).

In conclusion, the study revealed face and content validity of the HBSC item on food insecurity. It found that the proportion of people reporting food insecurity is valid, and the findings of same associations between food insecurity and negative outcomes to key items on same dimensions as revealed in the literature from western and Arctic indigenous populations give indirect support to the validity of the measure in the Greenlandic setting. Although further research on explanatory and mediating factors is needed, the study indicates that the HBSC measure is a reliable indicator of food insecurity in Greenlandic schoolchildren.
